# Insights into the Cardiac Phenotype in 9p Deletion Syndrome: A Multicenter Italian Experience and Literature Review

**DOI:** 10.3390/genes14010146

**Published:** 2023-01-05

**Authors:** Flaminia Pugnaloni, Roberta Onesimo, Rita Blandino, Carolina Putotto, Paolo Versacci, Angelica Bibiana Delogu, Chiara Leoni, Valentina Trevisan, Ileana Croci, Federica Calì, Maria Cristina Digilio, Giuseppe Zampino, Bruno Marino, Giulio Calcagni

**Affiliations:** 1 Medical and Surgical Department of Fetus, Newborn and Infant, Bambino Gesù Children’s Hospital, IRCCS, 00165 Rome, Italy; 2 Department of Pediatrics, Obstetrics and Gynecology, “Sapienza” University of Rome, Policlinico Umberto I, 00155 Rome, Italy; 3 Rare Diseases Unit, Fondazione Policlinico Universitario Agostino Gemelli, IRCCS, 00168 Rome, Italy; 4 Pediatric Unit, Fondazione Policlinico Universitario Agostino Gemelli, IRCCS, 00168 Rome, Italy; 5 Catholic University of Sacred Heart, 00168 Rome, Italy; 6 Multifactorial and Complex Diseases Research Area, Bambino Gesù Children’s Hospital, IRCCS, 00165 Rome, Italy; 7 Department of Cardiac Surgery, Cardiology and Heart and Lung Transplant, Bambino Gesù Children’s Hospital, IRCCS, 00165 Rome, Italy; 8 Medical Genetics Unit, Bambino Gesù Children’s Hospital, IRCCS, 00165 Rome, Italy

**Keywords:** 9p deletion syndrome, clinical genetics, congenital heart defects, genotype–phenotype correlation

## Abstract

Chromosome 9p deletion syndrome is a rare autosomal dominant disorder presenting with a broad spectrum of clinical features, including congenital heart defects (CHDs). To date, studies focused on a deep characterization of cardiac phenotype and function associated with this condition are lacking. We conducted a multicentric prospective observational study on a cohort of 10 patients with a molecular diagnosis of 9p deletion syndrome, providing a complete cardiological assessment through conventional echocardiography and tissue Doppler imaging echo modality. As a result, we were able to demonstrate that patients with 9p deletion syndrome without major CHDs may display subclinical cardiac structural changes and left-ventricle systolic and diastolic dysfunction. Albeit needing validation in a larger cohort, our findings support the idea that a complete cardiac assessment should be performed in patients with 9p deletion syndrome and should be integrated in the context of a long-term follow-up.

## 1. Introduction 

Chromosome 9p deletion syndrome (MIM #158170) is a rare autosomal dominant disorder due to allelic loss of the short arm of chromosome 9 (9p−), with a still uncertain prevalence. 

The first report of the syndrome dates back to Alfi and colleagues [1], who described two unrelated patients with partial deletion of the short arm of chromosome 9 sharing common clinical features including trigonocephaly, craniofacial dysmorphisms, and hypertonia. 

Since then, several case reports and case series have extended the amount of data regarding phenotype. Clinical features are highly heterogeneous and are mostly represented by developmental delay and intellectual disability (ID) [2,3,4]. Behavioral issues, autism spectrum disorder, and speech and language deficits represent additional features that may be associated with 9p deletion syndrome [2,5,6,7]. Moreover, patients often display hypotonia and craniofacial features including trigonocephaly, hypertelorism, upward-slanting palpebral fissures, midface hypoplasia, epicanthus, low-set ears with ear auricle abnormalities, narrow palate, and short/broad neck. Sex reversal often occurs in patients with this genetic condition, and ambiguous genitalia are present up to 70% of cases. [5,8,9,10,11,12,13,14,15].

Cardiac involvement is frequently reported in 9p deletion syndrome, with a prevalence ranging from 35% to 50% of cases [2]. In addition to the commonly encountered clinical features, patients may display congenital heart defects with various degrees of severity [2,3], and cardiac murmur may represent an early clinical sign to arise suspicion of cardiac anomaly. Congenital heart defects (CHDs) have been previously described in several case reports, with ventricular septal defect (VSD), atrial septal defect (ASD), and patent ductus arteriosus (PDA) being the most recurrent anomalies [2,3]. The study on the largest cohort of patients with 9p deletion syndrome dates back to 2008, when Swinkels et al. described the clinical phenotype of 13 individuals affected by this condition [2]. Moreover, at the moment, studies assessing a focused characterization of the cardiac phenotype associated with this condition are lacking, and myocardial function evaluation has never been performed before.

With the aim of gaining insight into the cardiac phenotype of 9p deletion syndrome, we report here the largest cohort of patients cytogenetically diagnosed since 2008 along with their cardiac phenotype and cardiac functional evaluation. Furthermore, we delineate the chromosomal breakpoints of each patient as means to identify a potential correlation between the genotype of patients and their cardiac phenotype spectrum. Moreover, to support the cardiac features identified in our study, a narrative literature review, focused on the cardiac features in 9p deletion patients over the last 10 years, is also provided. 

## 2. Materials and Methods 

### 2.1. Population Cohort 

This was a prospective observational study on a cohort of 10 patients with a molecular diagnosis of 9p deletion syndrome, recruited from three Italian centers with reputed expertise in rare diseases and pediatric cardiology disorders: Bambino Gesù Children’s Hospital, IRCCS, Fondazione Policlinico A Gemelli, IRCCS, and Pediatric Cardiology Unit of Sapienza University of Rome. 

Patients from all over Italy were referred to these three centers. Demographic data and echocardiographic exams were collected within the study period January 2019–May 2022. 

The inclusion criteria for the study were confirmed genetic diagnosis of 9p deletion syndrome with and without associated rearrangements, ability to perform a complete echocardiographic exam including tissue Doppler imaging examination, and acceptance of the informed consent. We excluded all patients without their own or parental consent and without a complete echocardiographic examination. 

The Ethical Committee of Bambino Gesù Children’s Hospital approved the study protocol, and written informed consent was obtained from patient or legal guardian. 

### 2.2. Control Cohort 

The control group consisted of healthy patients admitted to the pediatric cardiology units. Patients in the control group were studied using the same echocardiographic protocol for syndromic patients by a pediatric cardiologist (C.P.), and we included patients with a structurally normal heart and no echocardiographic parameters suggestive for systolic or diastolic dysfunction. Written informed consent was obtained from a patient or legal guardian. 

### 2.3. Clinical Assessment

All patients recruited were assessed by clinical geneticists (C.D.G., G.Z., and V.T.), a pediatrician with expertise in disability (R.O.), and pediatric cardiologists (G.C., P.V., and A.B.D.). 

Systematic patient interviews investigated family and personal history of genetic syndromes, metabolic disorders, known congenital heart defects, arrhythmias, systemic hypertension, cardiovascular medications, and previous cardiac surgery procedures. 

Physical examination included height, weight, and body mass index (BMI) at the moment of evaluation, and obtained data were compared with normative data [16]. BMI was defined according to recommendations of Centers for Disease Control and Prevention (CDC) on patients between 2 and 20 years old; otherwise, the World Health Organization’s guidelines were used in the definition of BMI of individuals over 20 years old [17].

Body surface area (BSA) was calculated according to Haycock formula [18]. Blood pressure was determined during routine cardiac evaluation by standard methods using an appropriately sized cuff with the patient in the seated position, with the arm and back adequately supported, and after 5 min of rest. Measures were averaged after three evaluations. Results were compared with normative values that were gender-, age-, and height-matched [16].

### 2.4. Echocardiography 

All echocardiographic and Doppler examinations were performed by experienced pediatric cardiologists (P.V., A.B.D., D.L., and A.D.R.) and one sonographer (F.C.). 

An Epiq7 or iE33 ultrasound machine (Philips Medical, Andover, MA, USA) with a 1–5 MHz transducer (X5-1) and a 3–8 MHz transducer (S8-3) was used for studying 9p deletion syndrome patients; A GE Vivid 9 (Medical Systems, Oslo, Norway) with M5S-D (bandwidth 1.5–4.5 MHz) and 6S-D (bandwidth 2.4–8.0 MHz) was used for the control group. 

Conventional echocardiographic evaluations were performed according to the recommendations of American Society of Echocardiography [19].

Through M-Mode evaluation, echocardiographic data from the long-axis parasternal view included left-ventricle (LV) internal diameter in diastole (LVIDd) and systole (LVIDs), thickness of the interventricular septum in diastole (IVSd) and systole (IVSs), and LV posterior wall thicknesses in systole (PWs) and diastole (PWd). 

Ejection fraction (EF) and fractional shortening (FS) of the LV were calculated using Teichholz’s M-mode formula. LV mass was calculated using the Devereux formula [20] and indexed to height ^2.7^ [21].

The long-axis parasternal view was also used to assess proximal aorta diameters at four levels (aortic annulus, Valsalva sinuses for the aortic root, sinotubular junction, and proximal segment of the ascending aorta) and left atrium to aorta ratio (LA/Ao). 

All echocardiographic measures were indexed for BSA. 

Pulsed-wave Doppler echocardiography was used to assess transmitral and transtricuspidal flow patterns from the apical four-chamber view with the sample volume placed at the mitral and tricuspid valves leaflets tips. Early (E) and late (A) diastolic velocities, deceleration time (DT), and E/A ratio were measured for mitral valve. 

Right-ventricle global systolic function was assessed through tricuspid annular plane systolic excursion (TAPSE). 

In addition to the conventional echocardiographic measures, tissue Doppler imaging (TDI) studies were performed from the four-chamber view, with Doppler sample volume placed at the lateral (lateral mitral annulus, LMA) and septal (septal mitral annulus, SMA) myocardial segments of the mitral annulus and at lateral border of the tricuspid annulus (tricuspid lateral annulus, TLA); peak systolic (S’), early (e’) diastolic, and late diastolic (a’) waves were measured for both ventricles from a minimum of three cardiac cycles and averaged. 

Isovolumetric relaxation time (IVRT), isovolumetric contraction time (IVCT), and ejection time (ET) were measured on LMA, and the LV Tei index was calculated using the following formula: (isovolumic relaxation time + isovolumic contraction time)/ejection time.

E/e′ ratios for the LV (septal and lateral segments) were determined using previously estimated Doppler transmitral values.

The acquired data were analyzed offline using dedicated software (GE Health Care Clinical Systems, EchoPAC—version 113, Horten, Norway, and Philips Lab Cardiac AnalyQsis—version 8 and 10, Philips Heathcare Inc., Amsterdam, the Netherlands.

### 2.5. Molecular Analysis 

The molecular cytogenetic analysis (array-CGH, SNP array) was carried out on DNA extracted from peripheral blood lymphocytes for the 10 patients with nomenclature used according to the International System for Human Cytogenomic Nomenclature 2016 (ISCN). Segregation analysis of the molecular defects was performed according to parents’ availability. 

Except for the two analyses of SNP array-CGH (patient 7 and 9), array-CGH was performed in all the other cases using the Invitrogen pure link Genomic DNA Mini Kit (patient 2) and Agilent kit 8 × 60K (patients 1, 3, 4, 5, 6, and 9). Data analysis was performed using software CytoSure Interpret Software v.4.10.35 (patient 2) and FE Agilent technologies (Santa Clara, CA, USA) (patient 1, 3, 4, 5, 6, 9).

SNP array-CGH was performed in patient 7 using CytoSNP-850K (Illumina, San Diego, CA, USA) and analyzed with the Bluegenome Bluefuse Multi Software Edition 4.4 software, while it was performed in patient 10 using the CytoScan HD and chromosome analysis suite v4.0 software.

To evaluate the genomic region involved, the array-CGH and SNP-array data were mapped on Decipher software (https://www.deciphergenomics.org/browser, assessed on 30 September 2022) and the UCSC genome browser (https://genome.ucsc.edu/cgi-bin/hgTracks?db=hg38&position=lastDbPos&pix=1687, accessed on 14 October 2022) to build GrCh38 of the human genome. 

Molecular results and genomic data for the 10 unrelated patients are shown in Table 1.

### 2.6. Statistical Analysis 

Stata 17.0 (StataCorp. 2021. Stata Statistical Software: Release 17. College Station, TX: StataCorp LLC) was used for the statistical analysis. Continuous variables were expressed as the mean ± SD (range). Qualitative variables were expressed as the frequency and percentage. When two groups were compared for echocardiographic parameters, the nonparametric Mann–Whitney U test was performed to identify significant differences. A value of *p* < 0.05 was considered statistically significant.

## 3. Results 

Demographic characteristics of the study cohort (n = 10) and the control group (N = 22) are summarized in Table 2.

The median age of patients in the study cohort was 18.8 years (IQR 5.5–25.5 years), and healthy patients had a median age of 17.0 years (IQR 12–18). There was no statistically significant difference in age, gender, weight, height, BMI, and BSA between the cohort patients and controls. 

At the time of evaluation, all patients had normal blood pressure. Moreover, none of them had a family history of CHDs or a personal history of arrhythmias, hypertension, or cardiovascular interventions. 

BMI was normal in 6/10 patients, while 3/10 patients were overweight (BMI > 25 kg/m^2^ or BMI > 85th percentile) and 1/10 was underweight (BMI < 5th percentile).

CHDs were found in 2/10 patients with 9p deletion syndrome (20%). As shown in Table 1, genomic coordinates were available for all patient except for patient 10.

Two septal defects were noted in our cohort. In particular, one patient showed an ASD, whereas the second patient displayed both ASD and VSD. No major CHDs were detected in our population, but 3/10 (30%) showed mild atrioventricular valve insufficiency. 

Two-dimensional, M-mode, and pulsed-wave Doppler conventional echocardiographic data of the patients and controls are listed in Table 3, whereas TDI findings are listed in Table 4.

LV mass and LV mass BSA-indexed were significantly smaller in the study group compared with controls (*p* = 0.040, *p* = 0.006, respectively). No difference in left-atrial and LV M-mode measurements was detected.

A significant difference in the aortic dimension indexed by BSA was found. In particular, patients with 9p deletion syndrome exhibit greater aortic annulus size/BSA, greater sinotubular junction size/BSA, and greater proximal ascending aorta size/BSA, despite showing normal values when aortic root Z-scores were obtained [22].

LV systolic function assessed through the EF and FS was similar between the two groups, whereas the TDI-derived parameter of LV myocardial systolic function (LMA S′) was significantly lower in the 9p deletion syndrome group. 

Moreover, echocardiographic data regarding LV diastolic function such as E/A, LMA e’, and SMA e’ were significantly lower in 9p deletion syndrome group than the controls, whereas E/e’ septal and E/e’ lateral values were similar in the two groups. 

Findings related to the systolic and diastolic function of the right ventricle did not differ significantly among the two groups; TAPSE and TLA TDI parameters were completely preserved in all groups.

## 4. Discussion

We conducted the first Italian case study focused on a global cardiac phenotype assessment in 10 patients with 9p deletion syndrome.

The 9p deletion syndrome is a rare entity mainly characterized by ID and craniofacial abnormalities such as midface hypoplasia, long philtrum and trigonocephaly. Albeit less frequently described, patients may also present more severe malformations such as abdominal wall defects (omphalocele), congenital diaphragmatic hernia (CDH), and CHDs [23]. 

Location and extension of 9p deletions can vary among affected individuals; therefore, several phenotypic characteristics, such as cardiac defects, are variably expressed [2,3]. 

While the proximal region (9p23–p22) is considered responsible for the clinical manifestation especially trigonocephaly, the distal region (9p23.3) is associated with autism, ID, delayed speech, behavioral problems, 46,XY reversed sex (female external genitalia), and ambiguous genitalia in both sexes [4].

With regard to non-cardiovascular aspects, deletion of specific genes on chromosome 9p has been related to the onset of well-defined phenotypic traits. According to the literature, *CER1* and *FREM1* have been suggested as responsible for trigonocephaly, *DOCK8* has been proposed as a candidate gene for mental retardation and seizures, *FOXD4* has been related to speech and language delay, and *DMRT1* may play a role in sex reversal [8,10,24,25,26].

However, no consensus has yet emerged about the critical region associated with CHDs in 9p deletion syndrome.

Previous studies on murine embryos reported *KDM4C* on 9p24.1 as a gene involved in cardiac differentiation [27,28], whereas *CER1* located on 9p22.3 seems to have a role in cardiac myogenesis [29]. Furthermore, mutations of *FOXD4* on 9p24.3 are associated with dilated cardiomyopathy [30]. Moreover, *DOCK8* is involved in the reorganization of the actin filament system and is expressed in many organs, including heart and brain; therefore, its implication in pathogenesis of CHDs is yet to be defined [10,13].

Variable deletions sizes and heterogenous chromosomal defects are associated with the haploinsufficiency of the 9p region. The critical region involved has been studied by different groups. Christ and colleagues [31] studied the cytogenetic breakpoints in 24 patients with 9p deletion syndrome and identified a critical region of about 8 Mb in 9p23–p22 between D9S286 and D9S285. Later studies [25,32] narrowed the candidate region for 9p deletion syndrome to a 3.5 Mb interval from RP11-933C16 to RP11-725C9, encompassing at least seven genes. In 2008, Swinkels and colleagues [2] further refined the critical region to 300 kb on chromosome 9p22.3.

Although several gene mapping studies within the deleted region have been extensively analyzed, genotype–phenotype correlation has not been established for this condition due to the frequent involvement of additional chromosomal rearrangements, as well as the lack of a consistent molecular characterization. Moreover, the disruption of 29 of the genes mapped in the region can potentially cause an unmasking of recessive traits and contribute to complex presentations [23].

Heart involvement is well known and extensively reported in genetic disorders. Aneuploidies, such as Down syndrome and Turner syndrome, are commonly associated with CHDs [33,34,35]. Similarly, peculiar cardiac phenotypes are associated with genetic conditions caused by single-gene variants including RASopathies, CHARGE, Kabuki, and many other syndromes [36,37,38]. The heart is also largely involved in a consistent number of rare disorders due to copy number variants (deletion or duplication) encompassing multiple genes with highly variable phenotypic expression [39].

Even though the cardiac phenotype, as part of the 9p deletion syndrome spectrum, has been reported since the first description of the syndrome by Alfi et al., the functional cardiac involvement is poorly described. Ref. [1] Since then, CHDs have been reported in several case reports worldwide, as can be appreciated in Table 5, which shows a brief literature review of the last 10 years.

Cardiac features in 9p deletion syndrome are highly heterogeneous and can manifest with a wide range of severity.

In our cohort, septal defects were the prominent features and were present in 20% (2/10) of patients, while an additional 30% (3/10) showed mild atrioventricular valve insufficiency. Our results concerning CHDs were comparable with those reported previously [4], even if, in our cohort, no major CHDs were found.

In detail, the present study showed that 9p deletion syndrome patients have significantly changed LV geometry. LV mass and LV mass indexed for BSA were significantly smaller in the 9p deletion syndrome group when compared to controls. A possible effect of cardiac exercise on these results may be speculated. Recently, Krysztofiak and coauthors, demonstrated that regular physical activity in children and young adolescents leads to progressive heart physiological changes that include increased LV myocardial thickness and LV mass [49]. 

Even though hypotonia and psychomotor development delay [23] may contribute to the different LV geometry, it is possible to argue that the cardiac phenotype might result by the combination of environmental elements (such reduction of physical exercise) and epistatic effects of genes deleted within the critical region.

Nevertheless, the prognostic significance of our findings remains unclear; in the adult population, higher LV mass/BSA has been proposed as an independent risk factor for sudden cardiac death [50] even though the same results in young adolescents are still controversial. Similarly, a relationship between structural changes in terms of lower LV mass index and myocardial function is unknown.

An additional interesting finding in our cohort is represented by aortic remodeling in terms of increased aortic dimensions when indexed for BSA.

Aortic dilation represents a well-documented entity among patients with specific syndromes (i.e., Marfan syndrome, Turner syndrome, and RASopathies) and has been associated with several CHDs [50], but there is no evidence reporting its presence or extent in patients with 9p deletion syndrome. Probably, we can postulate that our observation results from performing a complete echocardiographic study following a standardized protocol based on ASE guidelines.

Aortic dilatation may be dependent on primary aortic medial degeneration or a secondary result from hemodynamically significant volume overload. Medial degeneration is due to fragmentation of elastic fibers, and it is clinically associated with additional abnormal features of the skeleton and connective tissue systems (joint laxity, scoliosis, pectus excavatum, or pectus carinatum). In our cohort, none of these features were clinically evident, and aortic root measurements were found to be within normal limits when compared to normograms (Z-scores). Greater aortic dimensions/BSA in 9p deletion syndrome patients may be related either to the intrinsic properties of the aortic root or to hemodynamic abnormalities that deserve further investigation.

The clinical significance and potential complications of these results should be assessed through a follow-up program evaluating the possible progression of structural changes within the aortic structure. Nevertheless, we are fully aware that our results need to be validated in a larger sample cohort.

Our study also adds significant information regarding LV functional parameters concerning both systolic and diastolic function.

Recent studies have highlighted the value of cardiac function assessment with different echo modalities (TDI and Speckle tracking echocardiography) in syndromic patients [51]. In this regard, our study provides the first complete functional cardiac assessment in patients with 9p deletion syndrome.

In particular, we identified statistically significant differences in LV systolic and diastolic function compared with healthy controls, whereas RV performance remained normal. Furthermore, when using TDI parameters, 9p deletion syndrome patients showed lower S’ wave velocities for lateral mitral annulus and reduced LV diastolic performance when compared to the control group.

All patients in our cohort are asymptomatic; we should, therefore, speculate a subclinical systolic and diastolic dysfunction which can possibly evolve into progressive and symptomatic heart failure, suggesting the need of a cardiac follow up for these patients.

Commonly, obesity, hypertension, dyslipidemia, and altered insulin profile are well established cardiovascular risk factors in young adults [52].

Congenital hyperinsulinism and hyperinsulinemic hypoglycemia have been previously reported among patients with 9p deletion syndrome [51,52,53], and a recent study by Banerjee and colleagues refined the critical region for hyperinsulinemic hypoglycemia to a 7.2 Mb deletion, even if the genes responsible for this feature within this locus are still not known [46].

There is a well-known close relationship between dysregulated insulin secretion and heart failure [54]; however, in our cohort, insulin abnormalities were excluded. None of our patients were obese or had ever shown dyslipidemia or hypertriglyceridemia on routine laboratory tests. Moreover, all patients in our cohort displayed normal arterial blood pressures when compared to age-, gender-, and weight-matched controls, and none of them had ever suffered from hypertension.

Myocardial strain and strain rate parameters were not assessed in our population due to the poor collaboration of these subjects when a longer exam should be performed. However, we strongly suggest performing these measurements when possible because it may add valuable information about LV dysfunction in this cohort.

In our cohort, the patient with the most severe cardiac phenotype (ASD and VSD) carried the largest 9p deletion (17.9 Mb) among all the patients analyzed. Notably, all the other patients with even mild cardiac involvement (ASD, tricuspid insufficiency, and mitral insufficiency) had a concomitant complex rearrangement involving a second chromosomal region with at least 8 Mb in size.

None of the genes mapped within the deleted region are exclusively associated with myocardial function; therefore, a definite genotype–phenotype correlation is still missing, and a prospective study with larger population is warranted.

We are aware that our study shows some limitations mainly due to the limited sample size of our cohort. Our findings concerning both cardiac morphological and functional aspects have to be interpreted cautiously since such results come from a limited number of echo studies. We firmly believe that results validation in a larger cohort is necessary, and that cardiac assessment should not be underestimated and should be integrated in the context of a long-term follow-up.

Even though the presence of a cardiac phenotype has been consistently associated with the condition since the first report of 9p deletion syndrome (Table 5), the long-term clinical implications of the cardiac defects had not been defined yet. In this regard, our study represents the first report investigating the cardiac characteristics in a highly selected 9p deletion syndrome cohort, highlighting differences in cardiac function that deserve a longitudinal evaluation to assess a potential effect into adulthood.

## 5. Conclusions

The present study adds valuable information about cardiac features in the context of 9p deletion syndrome and demonstrates that even patients affected by this condition without major CHDs may display subclinical cardiac structural changes and LV systolic and diastolic dysfunction.

Conventional echocardiography- and TDI-derived parameters are clinically useful tools for global cardiac assessment of patients with 9p deletion syndrome, and we suggest that these parameters may be used in the future for personalized clinical assessment and follow-up.

## Figures and Tables

**Table 1:** Molecular data and cardiac phenotype of study population.

	Patient 1	Patient 2	Patient 3	Patient 4	Patient 5	Patient 6	Patient 7	Patient 8	Patient 9	Patient 10
**Gender**	female	male	male	male	male	female	female	female	male	female
**9p Deletion Coordinates (Grch38)**	(271,257–14,270,849) × 1	(204,090–11,086,066) × 1	(271,257–4,600,279) × 1	(271,257–4,210,335) × 1	(214,367–16,307,944) × 1	(271,257–3,972,331) × 1	(46,587–7,250,826) × 1	NA; 9 (p24.3p23) del	(271,257–15,041,024) × 1	(203,861–18,114,932) × 1
**A-CGH resolution**	75 kb	200 kb	75 kb	75 kb	75 kb	82 kb	SNP array: 5 kb	NA	41 kb	SNP array: 75 kb
**Deletion size (Mb)**	14	11	4.5	3.9	16	3.7	7.2	9.6	14.8	17.9
**Reciprocal duplication**	no	no	no	no	no	yes	no	no	None detected	no
**Segregation analysis**	Not available	Not available	Not available	De novo	Not available	Not available	Father: 46, XY, ish t(6;9) (q27-, p24+; p24-, q27+) (RP11-37D8-, RP11-143M15+; RP11-143M15-, RP11-37D8+)	Not available	Not available	Not available
**Other genetic anomalies**	No	No	No	9 (q22.11) del (68,522,531–68,729,391) × 1 206 Kb	No	9 (p24.2p21.3) dup (4,051,471–25,205,566) × 3; 21 Mb	6 (q26q27) dup (161,860,136–170,610,382) × 3; 8.6 Mb	7 (q36.1q36.3) dup, 7.1 Mb	16 (q23.2q24.3) dup (80,252,108–90,044,855) × 3; 9.8 Mb	No
**Current age (eayrs)**	5	18	21	27	32	19	25	10	4	2
**Cardiac phenotype**	No CHDs	No CHDs	No CHDs	No CHDs	No CHDs	MV and TV insufficiency	MV insufficiency	No CHDs	ASD	OS ASD, VSD

MV: mitral valve; TV: tricuspid valve; ASD: atrial septal defect; OS ASD: ostium secundum atrial septal defect; VSD: ventricular septal defect.

**Table 2:** Demographic characteristics of the study population.

	Patients with Del9p (n = 10)		Control Sample (n = 22)		*p*
	n	%	n	%	
**Female**	5	50.0	9	40.9	0.631
**Age (completed years)**					
Mean (SD)	16.5 (10.9)		15.2 (3.3)		0.403
Median (IQR)	18.8 (5.5–25.5)		17.0 (12–18)		
**Weight (kg)**					
Mean (SD)	50.6 (25.0)		50.3 (14.2)		0.917
Median (IQR)	55.5 (32.0–69.8)		47.8 (42–60.0)		
**Height (cm)**					
Mean (SD)	147.0 (32.3)		160.6 (16.1)		0.791
Median (IQR)	160.0 (136–167)		160 (150–169)		
**Body Surface Area (BSA, m^2^)**					
Mean (SD)	1.4 (0.5)		1.5 (0.3)		0.907
Median (IQR)	1.4 (1.3–1.7)		1.5 (1.4–1.7)		

**Table 3:** Conventional echocardiographic data in the study cohort and controls.

	Patients with Del9p (n = 10)				Control sample (n = 22)				*p*
	Mean	SD	Median	IQR	Mean	SD	Median	IQR	
Left ventricle end-diastolic diameter (LVIDd, mm)	40.2	9.1	42.0	32.0–46.0	45.7	4.4	43.5	43.0–48.0	0.076
Left ventricle end-systolic diameter (LVIDs, mm)	25.4	6.9	26.5	18.0–29.0	29.6	2.8	30.0	27.0–31.0	0.067
Septal diastolic diameter (IVSd, mm)	7.1	1.7	7.3	5.4–8.4	8.2	1.5	8.0	7.0–9.0	0.100
Septal systolic diameter (IVSs, mm)	10.6	2.1	10.1	9.3–12.5	11.7	2.0	11.0	10.0–12.5	0.297
Left ventricle posterior wall during diastole (PWd, mm)	5.8	1.2	5.6	5.1–6.2	6.7	1.4	6.0	6.0–8.0	0.099
Left ventricle posterior wall during systole (PWs, mm)	10.8	2.2	11.0	10.6–11.8	11.7	2.8	11.0	9.0–13.0	0.772
LVIDd/BSA (mm/m^2^)	31.7	5.5	30.8	27.7–36.3	18.4	13.1	25.0	4.7–28.9	**0.020**
LVIDs/BSA (mm/m^2^)	17.8	7.0	18.9	16.7–22.5	20.2	3.3	19.9	18.1–21.6	0.534
IVSd/BSA (mm/m^2^)	5.1	1.0	4.8	4.4–6.0	5.5	1.0	5.5	4.7–6.4	0.328
PWs/BSA (mm/m^2^)	4.3	1.1	4.1	3.6–4.5	4.5	0.7	4.4	4.1–5.0	0.217
Left atrium/Aorta	1.2	0.2	1.2	1.0–1.3	1.3	0.2	1.3	1.2–1.4	0.100
Left Ventricle Mass (LV mass, g)	84.3	47.1	81.6	53.2–105	126.1	55.7	106.7	86.5–141.1	**0.040**
LV mass/BSA (g/m^2^)	59.4	18.4	50.1	46.7–74.3	83.7	21.8	81.0	65.9–96.8	**0.006**
Aortic Anulus (Ao AN, mm)	16.6	6.5	18.5	13.2–21.2	16.4	2.8	15.3	14.2–18.0	0.464
Ao AN/BSA (mm/m^2^)	13.4	1.9	12.3	12.0–15.1	11.1	1.9	10.8	9.6–12.4	**0.008**
Aortic Bulb (Ao BULB, mm)	23.5	4.9	25.0	20.0–27.2	22.9	3.8	22.9	20.0–24.8	0.708
Ao BULB/BSA (mm/m^2^)	17.1	2.6	16.0	15.4–18.2	15.5	2.5	15.2	13.9–17.5	0.086
Sinotubular Junction (ST JUNCTION, mm)	20.5	4.6	22.0	20.0–22.9	18.8	3.5	18.1	16.0–20.0	0.214
ST JUNCTION/BSA (mm/m^2^)	14.9	1.5	14.7	13.4–15.7	12.7	2.2	12.3	11.3–13.9	**0.007**
Proximal Ascending Aorta (PROX AA, mm)	21.7	4.9	23.0	18.0–25.9	20.5	2.7	20.0	18.2–22.0	0.339
PROX AA/BSA (mm/m^2^)	15.8	2.1	15.2	14.2–16.4	13.9	1.9	13.7	12.5–14.6	**0.016**
Left ventricle Ejection fraction (EF, %)	65.9	6.4	64.2	60.6–71.0	64.3	3.4	64.5	63.0–67.0	0.787
Left ventricle Fractional Shortening (FS, %)	37.1	4.0	37.0	33.0–40.3	36.2	6.9	35.5	34.0–37.0	0.298
Mitral valve: Peak E (cm/s)	86.6	10.6	85.3	78.9–95.3	108.2	18.0	101.0	94.0–117	**0.002**
Mitral valve: Peak A (cm/s)	59.1	7.0	60.5	59.9–62.4	53.4	13.3	51.0	45.0–66.0	0.386
Mitral valve: Deceleration Time (DT)	81.3	66.2	104.0	7.3 –132.5	174.9	22.5	171.0	158.0–200	**<0.001**
E/A ratio	1.5	0.2	1.4	1.3–1.7	2.1	0.6	2.0	1.7–2.3	**<0.001**
Tricuspid annular plane systolic excursion (TAPSE, cm)	2.3	0.5	2.4	1.9–2.7	2.2	0.3	2.3	1.9–2.3	0.437

**Table 4:** TDI echocardiographic data in the study cohort and controls.

	Patients with Del9p (n = 10)				Control sample (n = 22)				*p*
	Mean	SD	Median	IQR	Mean	SD	Median	IQR	
**Left Ventricle**									
LMA S’ (cm/s)	9.2	1.8	9.1	8.3–9.7	23.3	36.2	12.0	12.0–15.0	**<0.001**
LMA e’ (cm/s)	15.5	1.4	16.0	15.3–16.3	37.6	56.1	20.0	19.0–25.0	**<0.001**
LMA a’ (cm/s)	8.5	2.3	7.4	7.3–9.3	13.0	20.2	7.0	5.0–8.0	0.230
SMA S’ (cm/s)	8.1	1.9	7.9	7.1–8.2	8.4	1.8	9.0	8.0–9.0	0.060
SMA e’ (cm/s)	12.7	2.3	11.9	11.0–14.8	16.3	1.9	17.0	15.0–18.0	**<0.001**
SMA a’ (cm/s)	7.6	1.4	7.2	6.7–8.5	6.7	0.9	7.0	6.0–7.0	0.101
LMA E/e’	5.6	0.6	5.5	5.2–5.9	4.7	1.5	5.2	4.2–5.8	0.152
SMA E/e’	6.8	1.5	7.0	5.4–8.2	6.7	1.3	6.8	5.5–7.2	0.792
Isovolumetric relaxation time (IVRT, ms)	59.4	19.1	61.0	53.0 –73.5	46.9	9.3	46.0	42.0–58.0	**0.026**
Isovolumetric contraction time (IVCT, ms)	56.2	20.6	59.5	39.0–70.5	50.8	11.9	47.0	42.0–60.0	0.337
Left Ventricle Ejection Time (ET)	298.4	88.8	282.5	255–306	308.5	22.0	312.5	292–323	0.076
TEI Index	0.4	0.1	0.4	0.4–0.5	0.3	0.1	0.3	0.3–0.4	**0.001**
**Right Ventricle**									
TLA: S’ (cm/s)	13.7	5.0	13.0	10.1–14.9	14.3	2.3	14.0	13.0–16.0	0.270
TLA: e’ (cm/s)	21.2	19.4	14.9	12.4–18.7	17.5	2.8	18.0	15.0–19.0	0.195
TLA: a’ (cm/s)	16.3	15.1	11.4	9.2 –14.9	9.4	3.1	9.5	7.0–10.0	0.195

LMA: lateral mitral annulus; SMA: septal mitral annulus; TLA: tricuspid lateral annulus.

**Table 5:** Literature review of the cardiac features associated with 9p deletion patients over the last 10 years.

Author, Year	Sex	Cardiac Phenotype	Karyotype	Chromosome 9p Deletion	9p Deletion Size	Other Genetic Anomalies	Technique Used	Reciprocal Duplication
Onesimo et al., 2012 [10]	M	PDA, bicuspid aortic valve, CoA	46,XY, del(9)(p24.1)	9p24.3p24.1 (204,367–6,582,172)	6.5 Mb	--	CGH-array	--
Recalcati et al., 2012 [40]	M	ASD type II	46,XY, der(9)del(9)(p22)dup(9)(p22p11.2).ish dup(9)(p13.2p11.2)	9p24.3p22.2(194,193–18,341,167)	18.3 Mb	- dupl(9)(p13.2p11.2) - dupl9p22.2p13.1	CGH-array	Inverted duplication: - dup(9)(p13.2p11.2)(RP11-3J10++,RP11-108C23++) - 9p22.2p13.1(18,368,491–39,884,938
Meloni et al., 2012 [41]	F	Tricuspid valve insufficiency with right atrium enlargement	Not performed	9p24.3p22.2(116,890–17,629,981)	17.6 Mb	- dupl20p13p12.1 (14.8 Mb)	FISH, MLPA, Whole-Genome Microarray	Chr20:8159–14,755,396
Spazzapan et al., 2016 [42]	F	ASD	46,XY,del(9p22)	Not reported	Not reported	--	Karyotype, FISH	--
	F	VSD	46,XY,del(9p22)	Not reported	Not reported	--	Karyotype, FISH	--
Gunes et al., 2017 [43]	M	VSD	46,XY,del(9p24)	Not reported	Not reported	--	Karyotype	--
León-Carlos et al., 2018 [44]	F	VSD	46,XX,der(9)t(2;9)(q31;p22)	Not reported	Not reported	Balanced translocation	Karyotype GTG-banding	--
Chai et al., 2020 [45]	M	Mildly PVS	46,XY,del(9)(p24.3)	204,193–611,620	407 Kb	- del9q34.3 (884 Kb) (chr9:140134632- 141018984) - dupl9q33.2q34.11 (5879 Mb)	CGH-array WGS	Chr9:124,664,562–130,543,964
Banerjee et al., 2020 [46]	M	VSD	46,XY,del(9)(p22.1)	0–19,200,000	Not reported		NGS	None detected
	F	ASD	46,XX,del(9) (p22.2)	0–17,600,000	Not reported		NGS	None detected
	M	ASD, PDA	46,XY,der(9)t(9;13)(p24;q22.3)	0–9,806,011	Not reported	Unbalanced translocation	NGS	Chr13:75,000,000–115,200,000
	F	ASD, PDA	46,XX,del(9) (p23).ish del(9)(pter-)	0–9,330,617	Not reported	--	NGS	None detected
	F	VSD	46,XX,der(9)t(8;9) (q24.1;p24)	0–7,800,000	Not reported	Unbalanced translocation	NGS	Chr8:117,800,000–146,400,000
	F	PDA, PFO/ASD type II	Not performed	0–7,200,000	Not reported	Unbalanced translocation	NGS	Chr7:0–10,000,000
Cordes Salby et al., 2021 [47]	M	PFO	46,XY,der(9)t(3;9) (p25.1;p24.3)pat	203,861–1,373,611	1.17 Mb	- dupl3p26.3p25.1 (13.5 Mb)	CMA	Chr3:61,891–13,562,132
	F	ASD	46,XX,der(9)t(3;9) (p25.1;p24.3)pat	203,861–1,373,611	1.17 Mb	- dupl3p26.3p25.1 (13.46 Mb)	CMA	Chr3:61,891–13,562,132
Mohamed et al., 2021 [4]	M	PDA	46,XY,del9p24.3p22.2	208,454_17,724,146	17.7 Mb	- del9p21.3 (1.1 Mb)	CGH-array	--
	M	PDA	46,XY,del9p24.3p22.2	Not reported	Not reported	--	CGH-array	
	F	ASD	46,XX,del9p24.3p22.2	208,454_16,880,694	16.8 Mb	--	CGH-array	
	F	VSD	46,XY,del9p24.3p22.2	(9p22) 17,380K 17,770K (9p22–23) 10,350k 10,780k	Not reported	--	CGH-array	
	F	ASD, PDA	46,XY,del9p24.3p22.2	(9p22) 17,380K 17,770K (9p22–23) 10,350k 10,780k	17.770 Mb	--	CGH-array	
	M	CoA	46,XY,del9)t(8;9)(p21;p23)	203,861_11,587,302	11.587 Mb	- dupl8p23.3p21.3(19 Mb)	CGH-array	Chr8:208048_19,434,723
Yao et al., 2022 [48]	F	Right ventricular wall thickening, aortic arch narrowing (fetal echocardiography)	46,XX,idic(4)(q11),der(9)t(4;9)(q11;p23)	9p24.3p23(208,454–10,039,391)	9.8 Mb	Unbalanced translocation	Karyotype, SNP-array	4p16.3p11(68,345–49,089,361) (49 Mb)

## Data Availability

The data that support the findings of this study are stored in Hospital database and are available from the corresponding author on a reasonable request.
